# Elevated expression of VEGFR-3 in lymphatic endothelial cells from lymphangiomas

**DOI:** 10.1186/1471-2407-7-105

**Published:** 2007-06-21

**Authors:** Susanne Norgall, Maria Papoutsi, Jochen Rössler, Lothar Schweigerer, Jörg Wilting, Herbert A Weich

**Affiliations:** 1 Department Gene Regulation and Differentiation, Helmholtz Centre for Infection Research, Braunschweig, Germany; 2 Department of Pediatrics 1, Georg-August-University, Goettingen, Germany; 3 Department of Pediatrics, Albert-Ludwigs-Universität, Freiburg, Germany; 4 Klinik für Kinder- und Jugendmedizin, Helios Klinikum Berlin-Buch, Berlin, Germany; 5 Dept. of Anatomy and Cell Biology, Georg-August-University, Goettingen, Germany

## Abstract

**Background:**

Lymphangiomas are neoplasias of childhood. Their etiology is unknown and a causal therapy does not exist. The recent discovery of highly specific markers for lymphatic endothelial cells (LECs) has permitted their isolation and characterization, but expression levels and stability of molecular markers on LECs from healthy and lymphangioma tissues have not been studied yet. We addressed this problem by profiling LECs from normal dermis and two children suffering from lymphangioma, and also compared them with blood endothelial cells (BECs) from umbilical vein, aorta and myometrial microvessels.

**Methods:**

Lymphangioma tissue samples were obtained from two young patients suffering from lymphangioma in the axillary and upper arm region. Initially isolated with anti-CD31 (PECAM-1) antibodies, the cells were separated by FACS sorting and magnetic beads using anti-podoplanin and/or LYVE-1 antibodies. Characterization was performed by FACS analysis, immunofluorescence staining, ELISA and micro-array gene analysis.

**Results:**

LECs from foreskin and lymphangioma had an almost identical pattern of lymphendothelial markers such as podoplanin, Prox1, reelin, cMaf and integrin-α1 and -α9. However, LYVE-1 was down-regulated and VEGFR-2 and R-3 were up-regulated in lymphangiomas. Prox1 was constantly expressed in LECs but not in any of the BECs.

**Conclusion:**

LECs from different sources express slightly variable molecular markers, but can always be distinguished from BECs by their Prox1 expression. High levels of VEGFR-3 and -2 seem to contribute to the etiology of lymphangiomas.

## Background

The blood vascular system supplies all organs with oxygen and nutrients while the lymphatic vasculature is crucial for the uptake of extra-cellular fluid, lipids from the gut and circulating immune cells during immune surveillance. Unfortunately, the lymphatics also serve as highways for metastatic tumour cells. Both vascular systems are anatomically and histologically closely related to each other, but they are also different as concerns their topography, architecture of their walls, and their cellular and molecular composition (reviews see [1-4]). In spite of the importance of lymphatic vessels in health and disease, e.g. 80% of carcinomas metastasize via the lymphatic system, they have received little attention until recently. This has been due to the absence of suitable markers that distinguish between lymphatic endothelial cells (LECs) and blood vascular endothelial cells (BECs). Recently, LEC markers have been discovered and characterized, including the hyaluronan receptor LYVE-1, the membrane glycoprotein podoplanin, the transcription factor Prox1 and the VEGFR-3 tyrosine kinase [5-8]. Use of these markers and the generation of new antibodies have permitted the isolation and characterization of relatively pure populations of BECs and LECs. The different isolation procedures described in the literature include fluorescence-activated cell sorting (FACS) and magnetic separation (MACS). Besides pan-endothelial molecules like CD31 (PECAM-1), the BEC marker CD34 together with LEC specific anti-podoplanin antibodies have been used for the separation of BECs and LEC from dermal cell suspension [9]. Some groups have successfully used micro-beads with antibodies against VEGFR-3, podoplanin or LYVE-1 [10-15]. However, the previous studies have used antibodies, which were not commercially available, and the primary LECs were derived from only one source, the foreskin of healthy neonates. Commercially available primary human dermal microvascular endothelial cells (HDMECs) are a mixture of BECs and LECs. Heterogeneity of microvascular ECs from other commercial sources (UtMVECmyo) has not been tested. Importantly, the molecular profile of LECs from diseased tissues, such as lymphangiomas, has not been characterized yet, which is an important diagnostic step toward treatment of the disease.

In the present study we investigated if LECs from different sources possess stable markers, and if primary cells from lymphangiomas overexpress molecules involved in the VEGF signalling pathway. Therefore, we compared a variety of blood and lymph endothelial markers by using commercially available antibodies, commercially available primary cells and LECs from two lymphangioma patients. Cells from lymphangiomas were isolated by a combination of "cell sweeping" and paramagnetic micro-bead selection. For the separation of commercially available primary cells, FACS and MACS sorting were used, and characterization of the cells was performed at RNA and protein levels. Our studies show that some LEC markers are variable whereas others, such as Prox1, can be used to distinguish reliably between LECs and BECs. Furthermore, our studies suggest that high levels of VEGFR-3 and -2 may contribute to lymphangioma formation.

## Methods

### Cell lines, tissue samples and cell culture

Primary human dermal microvascular endothelial cells (HDMEC) were purchased from Clonetics (neonatal pooled, Cambrex, Inc.) and from PromoCell (Promocell GmbH). Primary human umbilical vein endothelial cells (HUVECs) were from PromoCell, and primary human aorta endothelial cells (HAEC) and human myometrial uterine microvascular endothelial cells (UtMVEC-Myo) were from Clonetics/Cambrex. They were cultured in endothelial growth medium, EGM-2MV (PromoCell) or ECGM-MV2 (Clonetics).

LECs from lymphangiomas were isolated from two male patients, 10 and 4 months of age. Lymphangiomas were located in the axillary region of patient A, and in the upper arm of patient B. The studies were approved by the hospital's ethics committee and were performed by the informed consent of the patient's parents. Explants of endothelial cells were cultured over 4–6 weeks and, using the "cell sweeping" procedure two times per week, contaminating stromal cells were removed. This procedure has been described in detail before [16]. Explants, which did not appear to be pure after this procedure, were sorted with micro-beads coated with CD31 antibodies (see below) to remove contaminating fibroblasts.

LECs or mixed populations of LECs and BECs were cultured in the presence of 200 – 300 ng/ml dNdC-VEGF-C. All cells were grown on gelatinized plates and split 1:2 or 1:4 when they were confluent, and were used not longer than passage 7–8. Recombinant dNdC- VEGF-C was expressed and purified as described [17].

### Antibodies

We used mono- and polyclonal antibodies against human proteins. The mouse mAbs anti-CD31/PECAM-1 (WM59) and anti-CD34 (563) were purchased from BD/Pharmingen. Rabbit pAb anti-von Willebrand Factor was purchased from DAKO. The mouse mABs anti-Tie2 (clone 16) and anti-KDR/VEGFR-2 (3G2) were gifts from Dr. B. Barleon, Reliatech GmbH, as were the rabbit pABs anti-LYVE-1, anti-CD105/endoglin and anti-Prox1. Goat anti-mouse, goat anti-rabbit and CD31 micro-beads were purchased from Miltenyi Biotec. Mouse mAbs anti-VEGFR-3 (2E11 and 9D9F9 [18] and Tie1 (9F12) were gifts from Dr. K. Alitalo (University of Helsinki, Finland). Mouse mAbs anti-podoplanin and rabbit pAbs anti-podoplanin were gifts from Dr. D. Kerjaschki (Medical University, Vienna, Austria). Also mouse mAb anti-podoplanin/gp36 (18H5) was a gift from Dr. G. Zimmer (University of Veterinary Medicine, Hannover, Germany).

### FACS analysis and cell sorting

Confluent monolayers of ECs were dissociated by incubation with accutase (PAA). Cell pellets were re-suspended in PBS containing 2% FCS to a final number of 5 × 10^4 ^cells/sample, and incubated for 30 min on ice with 1 μg/ml anti-CD31, 2 μg/ml CD34, 5 μg/ml anti-podoplanin (G.Z.), 1:500 diluted anti-podoplanin (D.K.), 5 μg/ml anti-Tie1, 2 μg/ml anti-Tie2, 5 μg/ml anti-VEGFR-1/Flt-1, 5 μg/ml anti-VEGFR-2/KDR, and 4–6 μg/ml anti-VEGFR-3/Flt-4. Rabbit antibodies were used as follows: 1:1000 dilution for anti-podoplanin (D.K.), 1 μg/ml anti-LYVE-1 and 5 μg/ml anti-CD105/endoglin. Primary antibody binding was revealed with FITC-labelled goat-anti-mouse or anti-rabbit antibodies (Dianova GmbH). Negative controls were performed by omission of the first antibody. Dead cells were stained with propidium iodide. Cells were analysed by flow cytometry using FACS-Calibur and CellQuest software (BD). Overlays were executed with either CellQuest or Winmdi software (Joe Trotter, The Scripps Research Institute).

FACS sorting was done with a FACS-Vantage SE instrument using 0.5 -1 × 10^6 ^cells and the mouse-anti-podoplanin (G.Z.) and rabbit-anti-Lyve-1 antibodies. Besides goat-anti-rabbit FITC-conjugated also goat-anti-mouse PE-conjugated antibodies or rat-anti-biotin PE-conjugated antibodies were used for the isolation of double positive cells.

### Magnetic cell sorting

Magnetic labelling and separation was used for the isolation of LECs from lymphangiomas by positive sorting with CD31 paramagnetic micro-beads, if the cultures were contaminated with fibroblasts. For the isolation of LECs from human dermal microvascular endothelial cells, the antibodies mouse-anti-podoplanin (G.Z.) and rabbit-anti-Lyve-1 were used. For each separation, 0.2 – 1.5 × 10^6 ^cells were used and disaggregated by accutase as described for FACS analysis. For the separation process a MidiMACS separator with LS columns was used as recommended by the supplier (Miltenyi Biotec). A detailed isolation procedure for this method has been reported before [19]. After separation with micro-beads, cells were further cultured (> 8.000 cells/cm^2 ^in the presence of VEGF-C) for immunostaining and for FACS analysis.

### Immunofluorescence

For immunofluorescence-staining cells were directly seeded in 100 mm cell culture dishes or seeded in Lab-Tek II chamber slides (Nunc) coated with poly-lysine (5 μg/cm^2 ^in PBS) or with gelatine. Confluent cells were fixed 5 min with 4% paraformaldehyde. ECs were incubated for 15–30 min at room temperature in blocking buffer (1% bovine serum albumin in PBS) and permeabilization was achieved by incubation with 0.1% Triton X-100 for one minute. Cells were incubated 60 min with primary antibodies and 45 min with the secondary antibodies (FITC- or TRITC-labelled). Nuclei were counter-stained with 0.5 mg/ml 4',6-Diamidino-2-phenylindole (DAPI; Sigma).

### VEGFR-3 ELISA

The quantification of VEGFR-3 in cell lysates by ELISA has been described in detail before [20]. Briefly, confluent cells were homogenized in RIPA buffer (0.1% SDS, 1% Triton X100, 0.5% Na-deoxycholate, protease inhibitor cocktail in PBS) and protein concentrations were estimated according to a standard protocol (BCA assay, Perbio). Test samples were diluted at least 1:2 or 1:4 in dilution buffer. If necessary, higher dilutions were made. Each dilution was measured in triplicate.

### Micro-array analysis

Micro-arrays were performed at the micro-array core facility of the Medical Faculty of the Georg-August-University, Goettingen, Germany. We compared the expression profiles of LECs from each lymphangioma patient with that of HUVECs. Fluorescent dye-labelled probes for hybridization were produced with SMART fluorescence probe amplification kit (BD Biosciences). During reverse transcription of RNA, the resulting single stranded cDNA was elongated by introducing specific adaptor sequences at both the 5' and 3' ends. In an amplification step, a double-stranded cDNA was synthesized by PCR amplification to produce the amounts of probe necessary for hybridization (Eppendorf), using primers corresponding to the adaptor sequences. Following cDNA synthesis, aminoallyl-modified dUTP was incorporated into the cDNA during several rounds of primer extension. In a coupling step, N-hydroxysuccinimide-activated Cy3 and Cy5 dyes (Amersham Bioscience) reacted specifically with the modified dUTPs in the cDNA, producing labelled probes. The dye absorbance was measured with NanoDrop ND-1000 spectrophotometer to calculate the dye incorporation ratio per μg DNA. For hybridization of the probes, a mixture of Cy5 and Cy3 labelled probes was prepared. Per Array (44 k whole genome oligo micro-arrays; Agilent) 0.7 – 1.5 μg of Cy-labelled DNA was used. Hybridization was performed according to the Agilent '60-mer oligo micro-array processing protocol. Fluorescence intensities were measured with Agilent micro-array scanner. Normalization of the raw micro-array data was performed with non-linear loss regression. Differentially expressed genes were identified by ANOVA-procedure. The resulting p-values from the test statistics for significance were adjusted with the Benjamini-Hochberg method to control the False-Discovery-Rate.

## Results

### HUVECs and HAECs are negative for lymphatic endothelial markers

**Figure 1:**
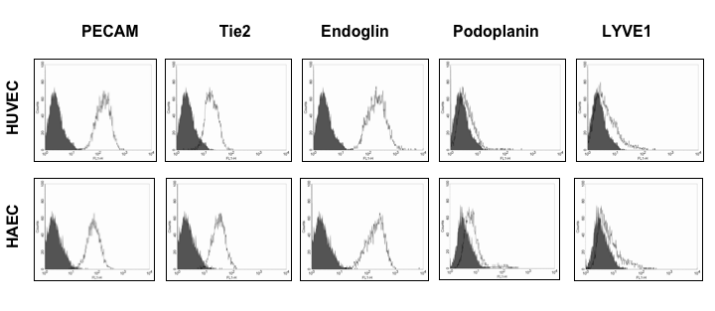
**Expression of blood and lymphatic markers on large vessel endothelial cells**. Typical profiles obtained by FACS analysis with the panendothelial markers CD31/PECAM-1, Tie2 and CD105/endoglin on primary HUVECs and HAECs (passage 3–5). Profiles obtained with lymphatic markers podoplanin and LYVE-1 on both of the control cell types are also indicated. The mean fluorescence intensities were normalized to the background fluorescence of the secondary antibody alone (grey).

**Figure 2:**
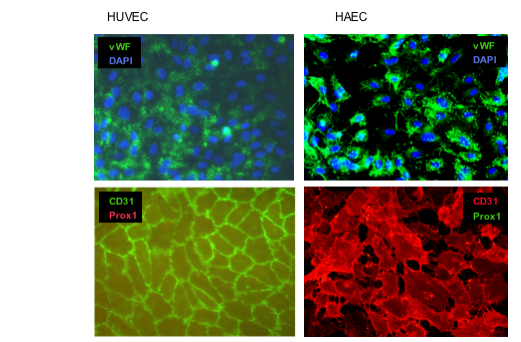
**Expression of von Willebrand factor (vWF), CD31/PECAM-1 and Prox1 in large vessel control cells**. Granular intracellular localization of vWF, membranous localization of CD31 and nuclear localization of Prox1 was detected by immunocytology in HUVEC and HAEC. Nuclei were counter-stained with DAPI. One representative staining out of several independent experiments is shown. Magnification, × 200.

As a negative control for our studies on LEC markers we have used two types of large vessel endothelial cells isolated from human umbilical vein (HUVEC) and aorta (HAEC). These cell types are from distinct blood vessels, either from venous or arterial localization, and are well characterized and commercially available. For FACS analysis we used the pan-endothelial markers CD31/PECAM-1, Tie-2 receptor and CD105/endoglin. Both vascular cell types were positive for the selected surface markers and there were only minor differences in the expression levels between the two cell types (Fig. 1). Antibodies against podoplanin have been used in several studies to isolate LECs [10,9], since podoplanin is a good marker for LECs and lymph vessels [21]. Besides podoplanin, LYVE-1 is a well-established marker for lymphatic vessels [5]. Only a minor subpopulation of HAECs expressed LYVE-1 at a low level (Fig. 1). Cell sorting with this polyclonal antibody is possible, but compared to podoplanin we often received a broader peak over a wider range, indicating a variable number of epitopes on the cell surface. However, only a small subpopulation of arterial and venous BECs were positive for LYVE-1. Von Willebrand factor is one of the best-characterized markers for endothelial cells. All cells from both types of large blood vessels were positive for this marker (Fig. 2). Also PECAM-1 stains all cell membranes from endothelial cells. The transcription factor Prox1 is one of the most stringent markers for LECs *in vitro *and for lymphatic vessels in tissue sections, and has been used for immunostaining since several years [7,22]. As expected, HUVECs and HAECs were negative for Prox1, showing that these are pure population of BECs (Fig. 2).

### Human dermal microvascular endothelial cells as a source for BECs and LECs

**Figure 3:**
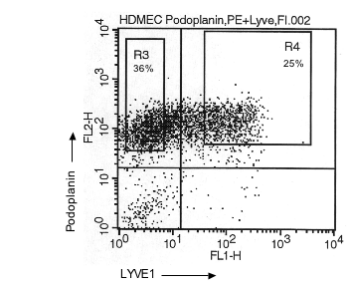
**Characterization of LECs isolated from dermal microvascular endothelial cells**. FACS sorting of HDMECs with the two antibodies against podoplanin and LYVE-1. Only podoplanin^+ ^cells are found in gate R3 and podoplanin^+^/LYVE-1^+ ^cells are found in gate R4. A total number of 9,6 × 10^5 ^cells from passage 6 have been used for cell sorting.

**Figure 4:**
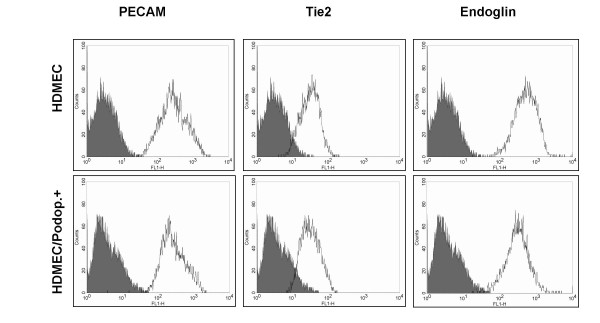
**FACS analysis of blood markers on dermal microvascular endothelial cells before and after podoplanin magneto-bead sorting**. Typical profiles obtained with the panendothelial markers CD31/PECAM-1, Tie2 and CD105/endoglin on primary HDMECs and on the same cells (passage 3–5) after podoplanin sorting with magneto beads.

**Figure 5:**
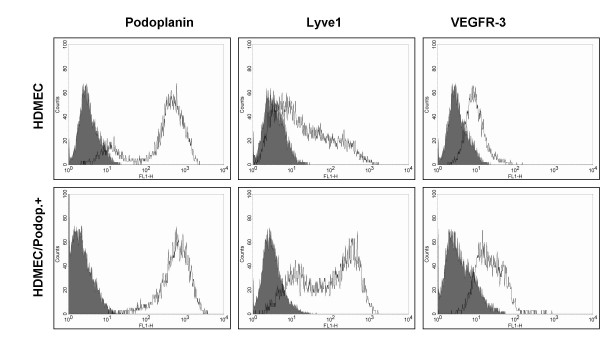
**FACS analysis of lymphatic markers on dermal microvascular endothelial cells before and after podoplanin magneto-bead sorting**. Typical profiles obtained with lymphatic markers podoplanin, LYVE-1 and VEGFR-3 before and after podoplanin-mediated cell sorting with a FACS sorter

Commercially available microvascular endothelial cells from foreskin of human neonates (HDMECs) have been used in recent years as a source for the isolation of LECs. These cultures contain a mixed population of BECs and LECs, and several protocols have been reported for the separation and culture of both types of vascular cells [19]. We have also used HDMECs from two different companies and tested two different separation methods with different antibodies in order to isolate LECs. First, we used micro-bead separation with podoplanin antibodies as reported recently [19]. This isolation method resulted in approximately 60–80% positive cells. Micro-bead separation with LYVE-1 antibodies is also possible, but results in only 15–40% of LYVE-1-positive cells. Similar results were found when FACS sorting was used. Here we applied the antibodies against podoplanin and LYVE-1 in combination (Fig. 3). A high percentage of the cells were positive for podoplanin and a substantial part of the population was podoplanin^+^/LYVE-1^+ ^(Fig. 3). An unexpected high percentage of LECs was found in a recent study when the same primary cells were used for characterization [23]. The podoplanin^+^, LYVE-1^+^, and podoplanin^+^/LYVE-1^+ ^cells were further cultured and analysed for LEC markers. A typical profile is shown in Fig. 4 + 5. As expected, podoplanin^+ ^cells showed an increase in the two LEC markers LYVE-1 and VEGFR-3. However, this increase was moderate and not all podoplanin^+ ^cells were also positive for LYVE-1 and VEGFR-3 (Fig. 5). Subcultures of podoplanin^+^, LYVE-1^+ ^or double-positive cells studied by FACS analysis revealed a certain percentage of cells negative for the surface markers, which were initially used for sorting. This may indicate that these cells down-regulate the expression levels of LYVE-1 and VEGFR-3.

**Figure 6:**
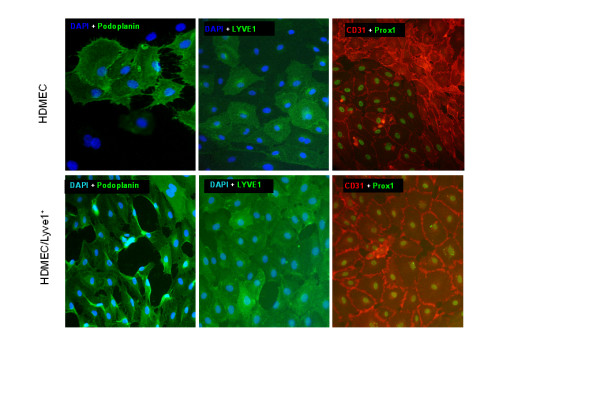
**Expression of CD31/PECAM-1, LYVE-1 and Prox1 in dermal microvascular endothelial cells before and after LYVE-1 sorting**. Membranous localization of CD31, LYVE-1 and podoplanin, and nuclear localization of Prox1 was detected by immunocytology in HDMECs. Nuclei were counter-stained with DAPI in some specimens. One representative staining out of several independent experiments is shown. Magnification, × 200.

We next assessed the expression of the transcription factor Prox1, as one of the most reliable markers for LECs [1,12]. Immunofluorescence was used to analyse Prox1 expression at the protein level. Most of the primary cells from foreskin (passage 4–7) were already positive for this marker, and after LYVE-1 or podoplanin selection almost 100% of the cells were positive for Prox1 and CD31 (Fig. 6). These cells could also be stained with LYVE-1 and podoplanin antibodies (Fig. 6).

### Myometrial microvascular endothelial cells are positive for LYVE-1, but negative for podoplanin and Prox1

**Figure 7:**
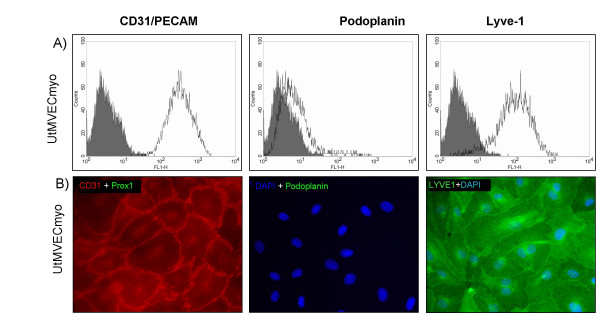
**Expression of lymphatic markers on UtMVECmyo**. **A: **Typical FACS analysis profiles obtained with the panendothelial marker CD31/PECAM-1 and the lymphendothelial markers LYVE-1 and podoplanin. **B: **Immunostaining for CD31, Prox1, podoplanin and LYVE-1. Nuclei were counter-stained with DAPI. One representative staining out of several independent experiments is shown. Magnification, × 200.

Normal human uterine microvascular endothelial cells isolated from the myometrium (UtMVECmyo) are commercially available. We followed the question are these cells also a mixture of BECs and LECs. Results obtained with this cell type were surprising. Cultured cells were positive for LYVE-1, but negative for podoplanin (Fig. 7). Furthermore, immuno-fluorescence was used to analyze Prox1 expression *in vitro*. Prox1 was undetectable, like podoplanin, whereas more than 90% of the cells were positive for LYVE-1 as estimated by DAPI counter-staining.

### Lymphatic endothelial cells isolated from lymphangiomas

**Figure 8:**
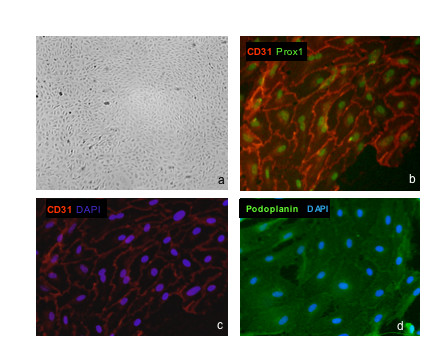
**Isolation and characterization of LECs from lymphangiomas**. Lymphendothelial cells (LECs) from lymphangioma explants isolated by „cell sweeping” were photographed under phase contrast microscopy. **a**) The cells from patient-A show the typical cobblestone morphology and are contact inhibited (passage 7, magnification, × 40.). Expression of CD31/PECAM-1 together with Prox1 in LECs (**b**). Membranous localization of CD31 and nuclear localization of Prox1 was detected by immunocytology. Magnification, × 200. **c**) Same specimen as in **b**) showing CD31 expression and nuclear counter-staining with DAPI. Magnification, × 200. **d**) Podoplanin staining. Nuclei were counter-stained with DAPI. One representative staining out of several independent experiments is shown.

**Figure 9:**
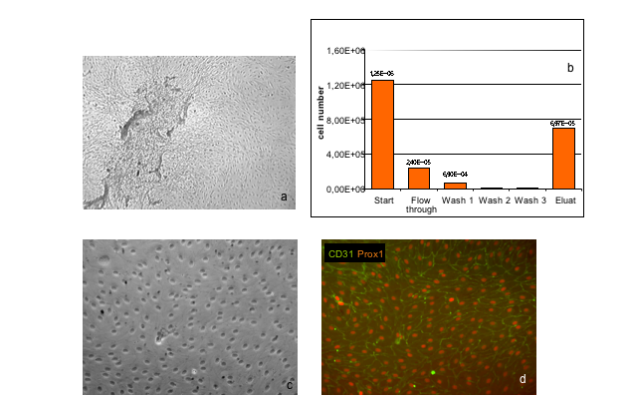
**Isolation and characterization of LECs from lymphangiomas**. LECs from lymphangioma explants (patient-B) isolated by CD31 microbead sorting shown by phase contrast microscopy. The culture was contaminated with stromal cells **(a**). Isolation of LECs with CD31 micro beads. Indicated are cell numbers after each step.(**b**). Cells are further cultivated and displayed the typical cobblestone morphology (**c**). **d**) Same specimen as in **c**) stained for CD31 and Prox1. Magnification: × 40 in (a) and × 100 in (c) and (d).

In the current study we had the unique possibility to isolate and characterize LECs from lymphangiomas of two young children. Both patients suffered from lymphangioma in the axillary and upper arm region. Both methods, "cell sweeping" and CD31 micro-bead sorting of mixed cell populations have lead to pure populations of LECs, about 4 – 6 weeks after surgical removal of the lymphvascular malformation. The cells from patient A had a cobblestone characteristic and microscopic morphology very similar to other primary endothelial cells (Fig. 8). Cells from patient B looked very similar (Fig. 9). The cells were cultured with the same medium as for microvascular ECs, but VEGF-C was added to the medium. This mitogen is specific for LECs [19] and helps to maintain cell viability and cell type characteristics under culture conditions.

**Figure 10:**
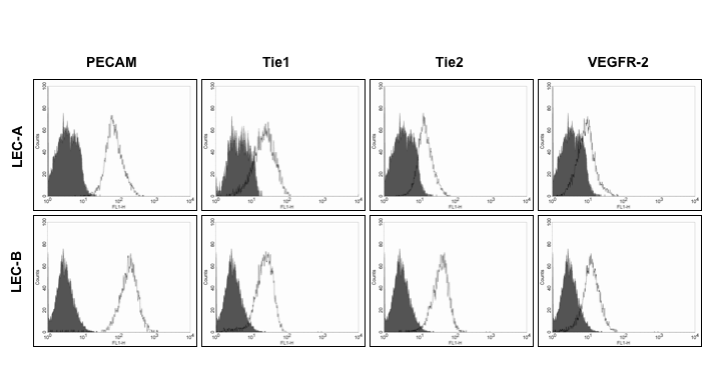
**FACS analysis of blood vessel markers on LECs from lymphangioma patients**. Expression of blood endothelial markers. Typical profiles obtained with the panendothelial markers CD31/PECAM-1, Tie1, Tie2 and VEGFR-2 on primary LECs from the two patients A and B (passage 4–6). All of the markers are expressed.

Expression of lymphatic markers was further analyzed by immunological methods. All isolated cells from patient A were CD31^+ ^and Prox1^+^. Furthermore, these cells could also be stained with podoplanin antibodies. They showed a very homogenous expression for podoplanin (Fig. 8d). Cells from patient B were generated similarly by "cell sweeping " and sorting with CD31 microbeads (Fig. 9). Staining with Prox1 confirmed that all cells possessed LEC characteristics (Fig. 9d). We next assessed the expression of a variety of EC markers by FACS analysis. We found that besides PECAM1 they were positive for Tie1, Tie2 and VEGFR-2 (Fig. 10).

**Figure 11:**
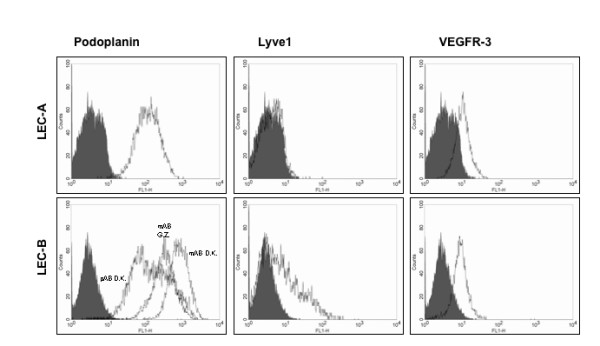
**FACS analysis of lymph vessel markers on LECs from lymphangioma patients**. Expression of lymph endothelial markers on LECs from lymphangioma patients. Typical profiles obtained with the markers podoplanin (three different antibodies for LEC-B), LYVE-1 and VEGFR-3 on primary LECs from the two patients A and B (passage 4–6).

We also tested three different antibodies against podoplanin. All of them gave a positive result. However, in contrast to the dermal LECs, LECs from lymphangiomas were either negative for LYVE-1 or expressed this marker only at low levels (Fig. 11). These results could be confirmed by immunofluorescence (not shown). Both of the LEC populations were positive for VEGFR-3, but expression appeared to be relatively low. However, the results obtained with the VEGFR-3 antibodies in the FACS analyses were variable. We therefore quantified VEGFR-3 expression with a recently developed sandwich ELISA that uses different VEGFR-3 antibodies [20]. The amount of VEGFR-3 protein in the lysates from large blood vessel endothelial cells was very low (1.4 – 2.7 ng/mg). UtMVECmyo were almost negative for VEGFR-3 protein (1.6 ng/mg), indicating that they are BECs rather than LECs, with the peculiarity, that they are positive for LYVE-1. In the lysates prepared from LECs originating from LYVE-1 sorting of HDMECs, VEGFR-3 protein concentrations increased to 19.3 ng/mg, while LECs from lymphangiomas reached values of 29.5 – 40.8 ng/mg, which was significantly higher than the concentrations found in foreskin LECs (Table 1).

**Table 1:** VEGFR3 quantification in cell lysates by ELISA

**Cell type**	**Total protein [mg/ml]**	**Concentration [ng/ml]**	**Concentration [ng/mg protein]**
*HUVEC*	0.67	1.77	2,65 +/- 0.06
*HAEC*	1.09	1.51	1.38 +/- 0.03
*UtMVECmyo*	1.03	1.64	1.60 +/- 0.01
*HDMEC*	0.36	2.78	7.73 +/- 0.61
*HDMEC Lyve1+*	0.53	10.25	19.34 +/- 0.75
*LEC A*	0.60	17.71	29.51 +/- 2.25
*LEC B*	0.42	17.15	40.84 +/- 1.66

Protein levels of VEGFR3 in various endothelial cell types measured by ELISA. Cell lysates were prepared from confluent cells and after dilution lysate samples were measured in triplicates. Indicated is the mean of VEGFR3 protein concentration in ng per mg of total protein (+/- SD).

For a broader characterization of the LECs from the two lymphangioma patients, we studied their gene expression profile in comparison to HUVECs (Table 2). The studies confirm the lymphendothelial origin of the cell, since we found expression of a large number of molecules that have previously been found by others in dermal LECs [12-14]. This applies to markers such as Prox1, podoplanin, reelin, c-Maf, macrophage mannose receptor and TIMP 3, as well as to growth factors and receptors such as FGF-12, TGF-α, neuropilin-2, integrin-α 9 and integrin-α1. Additionally, we found a large number of highly expressed LEC genes in the whole genome micro-arrays, which have not been detected previously, due to limitations of the micro-arrays used (data not shown). However, the levels for Lyve-1 and VEGFR-3 were not as high as could have been expected. The RNA data for Lyve-1 were in agreement with the variable results of the FACS analyses, however, the RNA data for VEGFR-3 were lower than could have been expected from the ELISA data, indicating high stability of the protein. In patient-B, we found very high levels of VEGFR-2 in the lymphangioma LECs, and both expression of VEGFR-2 and VEGFR-3 could be confirmed by immunostaining of tissue sections (data not shown). Additionally we observed expression of the adapter protein shc, phospholipase-Cγ (PLCγ) and p44 MAP kinase, which are involved in VEGFR-2 and -3 signal-transduction (review: 2). The data suggest that signalling via VEGFR-2 and -3 may be involved in lymphangioma formation.

**Table 2:** Expression profile of lymphangioma LECs compared to HUVECs using Agilent gene micro-arrays

**Molecule**	**Patient A**	**P-Value**	**Patient B**	**P-Value**
**LEC markers**				
Prox1	5.21	3.2779e-05	5.34	3.0458e-05
	5.00	0.000114	5.47	8.7549e-05
Podoplanin	7.26	0.000538	6.23	0.000847
Reelin	5.64	0.000634	5.12	0.000844
c-Maf	4.45	0.000177	5.17	0.000113
Macrophage mannose receptor	3.96	0.000157	4.48	0.000109
TIMP 3	5.60	2.3392e-05	5.80	2.1012e-05
Lyve-1	2.75	0.001547	1.89	0.004617
VEGFR-3	1.73	0.009537	2.38	0.003869
	2.54	0.000704	3.30	0.000323
**Growth factors and receptors**				
VEGFR-2	1.91	0.005006	4.18	0.000506
	*1.33*	*0.085223*	4.08	0.004490
Neuropilin-2	1.59	0.000646	2.89	0.000107
	1.47	0.001513	3.34	0.000133
	1.19	0.009847	3.09	0.000614
FGF-12	1.56	0.001699	2.67	0,000344
	0.98	0.005359	2.59	0,000304
TGF-α	5.03	0.000273	5.32	0.000230
Integrin-α9	2.46	0.000592	2.35	0.000677
	1.15	0.002815	1.70	0.000912
Integrin-α1	4.22	0.003322	2.69	0.011953
**VEGFR-2 and -3 signalling**				
SHC	2.34	0.000219	3.20	8.6303e-05
	1.73	0.000303	2.50	0.000100
PLCγ-1	1.80	0.000291	0.69	0.004961
PLCγ-2	n.r.	-	1.27	0.005680
MAPK3/ERK1	0.40	0.029350	1.63	0.000559
MAPK1/ERK2	-0.84	0.005289	-0.62	0.012707
	-0.45	7.9235e-06	-0.21	8.2757e-05
NF-kappaB1	0.27	0.026460	0.85	0.000982
	0.29	0.047104	1.16	0.001010

The up-regulation is expressed as the log_2 _ratio as compared to HUVECs. For MAPK1 we observed down-regulation. All values (except one written in italic) are statistically significant. Double values refer to transcription variants of the gene. n.r. = not regulated.

## Discussion

The main functions of the lymphatic vascular system are to maintain the fluid balance in the interstitial spaces, to provide a highway for circulating leukocytes, and to take up and transport larger particles such as chylomicrons and bacteria. These important functions of lymphatic vessels in health and disease are well known, but have rarely been studied experimentally (review: [4]). Malformations of lymphatic vessels, such as lymphangiomas, are associated with a failure in lymph transport and high morbidity of the patients [24]. The etiology of lymphangiomas is unknown, which may not be surprising, because even the mechanisms of normal embryonic development of lymphatic vessels are still a matter of debate (review: [2]). Some authors assume that lymphatic vessels are exclusively derived by sprouting from the venous system, whereas others suggest that there is an additional origin from mesenchymal lymphangioblasts [25]. A few "lymphangiogenesis" genes have been identified in patients presenting with congenital lymphedema, due to hypoplasia or dysplasia of the lymphatic vascular system. Milroy lymphedema in some, but not all, affected families is due to mutations in the VEGFR-3 gene [26,27], which encodes the receptor for the lymphangiogenic growth factors VEGF-C and -D [3]. Lymphedema-distichiasis is linked to the forkhead transrciption factor FOXC2 [28], and the lymphedema-hypotrichosis-telangiectasia syndrome is caused by mutations in the transcription factor SOX18 [29]. However, besides lymphangioma, there are approximately 40 syndromes that are associated with abnormal development of the lymphatic vascular system [30]. The isolation and characterization of LECs from normal and malformed tissues will facilitate the characterization of the diseases, as a first step for diagnosis and therapy.

### Isolation and characterization of LECs

Isolation studies have concentrated on LECs from foreskin and not from other tissues. Characterization of LECs from different healthy or diseased tissues has not been carried out. We have started to address this question and isolated LECs from normal and diseased tissues with commercially available antibodies. The expression and distribution of blood and lymph vessel markers was analyzed at RNA level with micro-arrays and at protein level by FACS analysis, immunocytology and ELISA. The main results of our studies show that

(i) LEC markers vary among the types of tissue used for cell culture

(ii) microvascular endothelial cells from the myometrium are a homogenous population of BECs, but positive for LYVE-1

(iii) LECs from lymphangiomas compared to those from foreskin have numerous markers in common, but show decreased LYVE-1 and increased VEGFR-3 expression

(iv) Prox1 is by now the most reliable marker of LECs.

Our data are in line with and extend previous studies demonstrating that LECs can be isolated from neonatal foreskin and identified by their ability to express markers like VEGFR-3, podoplanin and LYVE-1 [5,13,14].

Human dermal microvascular endothelial (HDMECs) have a high capacity to express lymphatic markers, and we observed that BECs seem to make up the minority of cells after primary isolation. This has also been observed in studies where these cells were used for immortalization [23]. It is not known by now, if directly after the initial isolation of the cells from foreskin with anti-CD31 antibodies by the supplier LECs are the numerical dominant cell type, or if during further expansion and splitting of the cultures the population of LECs preferentially increases. In initial experiments with HDMECs we found that the percentage of podoplanin^+ ^cells is very high (60–90%). Further characterization of the cells by FACS analyses showed that some batches can be divided into three groups: podoplanin-negative, moderately positive and strongly positive. However, most of the batches contain only two groups: podoplanin^- ^and podoplanin^+ ^(data not shown). Approximately 50% of the podoplanin^+ ^cells are also positive for LYVE-1, and these are the same cells, which are also positive for VEGFR-3. At the moment it remains unclear, if podoplanin^+^/LYVE-1^- ^cells represent an intermediate cell-type, and if only the podoplanin^+^/LYVE-1^+^/VEGFR-3^+ ^cells can be regarded as primary LECs from neonatal foreskin.

Growth of cultured LECs depends on the presence of VEGF-C, which, in mixed cultures, is supplied by the BECs [3]. After separation of LYVE-1^+ ^or podoplanin^+ ^cells we cultured them in the presence of VEGF-C as recommended before. However, selective stimulation of VEGFR-2 by VEGF-E is obviously also sufficient to grow and expand LECs in culture [31]. The activity profile of another family of endothelial growth factors, the angiopoietins (e.g. Ang-1 and Ang-2) is still controversially discussed. Angiopoietins bind the Tie2 receptor, and may activate or inhibit signal transduction in a cell-type and tissue-specific manner [32,33]. Angiopoietins are essential for the hierarchical organization of the blood vascular tree, but the expression of Tie2 in lymph vessels of the human has remained unclear. We have observed Tie2 expression in LECs sorted with anti-podoplanin antibodies from HDMECs, as well as Tie1 and Tie2 in LECs from lymphangioma patients. Mice deficient in Ang-2 show defects in the patterning and function of the lymphatic vasculature, and a lack of lymph nodes, and they develop chylous ascites [33]. Overexpression of Ang-1 in the skin of adult mice induces lymphangiogenesis, which is associated with VEGFR-3 up-regulation in LECs. Interestingly, proteolytically processed VEGF-C, which binds VEGFR-2 and -3, induces Ang-2 expression in LECs *in vitro *via VEGFR-2 signalling, indicating a new interaction between the VEGF and angiopoietin family members, which may regulate the hierarchy of the lymphvascular tree [34].

### Lymphangioma endothelial cells

Lymphangiomas are disfiguring neoplasias of childhood and may also manifest or enlarge rapidly in adulthood [35]. More than 95% of lymphangiomas occur in the soft tissues of the head, neck and axilla, with less than 5% occurring in the abdominal cavity. The prevalence of lymphatic malformations is 1.2 – 2.8‰ [36]. Macroscopically lymphangiomas are solitary, multicystic masses. The lining of the cysts is smooth and they have thin walls. Histological criteria for lymphangiomas are: 1) lymphatic spaces lined by endothelium, 2) fascicles of smooth muscle in the septa, and 3) lymphoid aggregates in the delicate collagenous stroma [37]. Immunoelectron microscopic studies have demonstrated up-regulation of CD31 and CD34 and show type IV collagen expression in lymphangiomas [38]. The vascular endothelial marker PAL-E is confined to blood vessels in lymphangiomas. VEGFR-3 mRNA has been localized to lymphangioma LECs [8]. The detection of transcripts for VEGF-C, VEGFR-2 and VEGFR-3 in endothelial cells from different lymphangiomas by in situ hybridization was reported before [39]. No expression of these genes was found in adjacent tissue or in normal lymphatic vessels.

With immunohistological methods, we have recently been able to identify VEGFR-3 and Prox1 in CD31-positive LECs of lymphangiomas [22].

Here we have measured the amount of VEGFR-3 protein in lysates from different endothelial cell types and found significantly higher expression in LECs derived from the two lymphangioma patients as compared to foreskin LECs. Significant amounts of VEGFR-3 and its intra-cellular signalling cascade (shc, PLCγ, p44MAPK) were also detectable at RNA level. Increased VEGFR-3 signalling may be a major course for aberrant lymph vessel formation since the opposite, lymphatic hypoplasia, can be observed in VEGFR-3 mutated patients with Milroy's disease [26,27]. The reasons or regulatory pathways for VEGFR-3 up-regulation are not known. Direct (mutations) or indirect mechanisms have to be considered, e.g. an increase of Ang-1 expression, as discussed above. However, we did not detect Ang1 or Ang2 expression in lymphangioma LECs at significantly higher levels as compared to HUVECs. Because lymphangioma tissue mainly consists of LECs and stromal cells, the ligands for VEGFR-3, VEGF-C and VEGF-D, may be derived from either of the cell types, acting in an auto- or paracrine mode, respectively. However, we have not found any measurable VEGF-C protein in the conditioned media obtained from stromal cells of the two patients. Also, we did not observe significant levels of VEGF-C and -D in the LECs. Other growth factors may be involved in the development of lymphangiomas. We have observed high expression of FGF-12 and TGF-α in lymphangioma LECs, but the significance of this finding remains to be studied. In contrast to BECs, LECs seem to be highly responsive to stimulation with hepatocyte growth factor (HGF) [15], but we could not detect significant expression of the HGF receptor, c-Met, in lymphangioma LECs as compared to HUVECs. We have found high levels of VEGFR-2 in the LECs from the lymphangioma patients as compared to HUVECs (Table 2). This is a surprising finding because usually VEGFR-2 mRNA levels of BECs and LECs are equal, or they are lower in LECs [12,13]. We did not find significant mRNA levels of the ligands PlGF, and VEGF-B in the LECs and only in patient-A there was high expression of VEGF-A, which may indicate an autocrine loop. Further studies with the cells *in vitro *and *in situ *will be necessary to identify the underlying mechanisms involved in LEC hyperplasia and dysplasia.

## Conclusion

We have developed methods to isolate LECs from normal dermis and lymphangioma tissues. The transcription factor Prox1 is a stable marker of LECs, whereas surface markers are regulated and variable. Characterization of lymphangioma LECs suggests an involvement of VEGFR-3 and -2 in the etiology of the disease. Gene array analyses have revealed large numbers of molecules involved in the regulation of growth, differentiation and function of LECs, and call for further functional characterization.

## Abbreviations

Ang-1, angiopoietin-1; Ang-2 angiopoietin-2; BEC, blood endothelial cell; EGM-2MV, endothelial growth medium microvascular ; ECGM-MV, endothelial cell growth medium-microvascular; FACS, fluorescence activated cell sorting; HA, hyaluronan acid; HAEC, human aortic endothelial cell; HDMEC, human dermal microvascular endothelial cell; HGF, hepatocyte growth factor; LEC, lymph endothelial cell; MACS, magnetic cell sorting, UtMVEC-myo, human myometrial uterine microvascular endothelial cell; PAL-E, Pathologische Anatomie Leiden-endothelium; Tie1, tyrosine kinase receptor 1; Tie2, tyrosine kinase receptor 2,

## Competing interests

The author(s) declare that they have no competing interests.

## Authors' contributions

SN and MP contributed equally to this work. JW and HAW designed the research, analyzed the data and wrote the paper; SN and MP performed the research, analyzed the data, wrote parts of the paper; JR provided primary tissue samples and LS conducted general supervision and bioinformatics. All authors read and approved the final manuscript.

## Pre-publication history

The pre-publication history for this paper can be accessed here:


